# Comparisons of chromosome Y-substituted mouse strains reveal that the male-specific chromosome modulates the effects of androgens on cardiac functions

**DOI:** 10.1186/s13293-016-0116-4

**Published:** 2016-11-23

**Authors:** Samantha D. Praktiknjo, Sylvie Picard, Christian F. Deschepper

**Affiliations:** 1 Institut de recherches cliniques de Montréal (IRCM) and Dept of Medicine, Cardiovascular Biology Research Unit, Université de Montréal, 100 Pine Ave West, Montreal, QC H2W 1R7 Canada; 2 Present address: Berlin Institute for Medical Systems Biology (BIMSB), Max Delbrück Center for Molecular Medicine (MDC) in the Helmholtz Association, Robert-Rössle-Str. 10, D-13125 Berlin, Germany

**Keywords:** Male-specific portions of chromosome Y, Chromosome Y substituted mouse strains, Genetic variants, Circadian rhythms, Circadian genes, Androgens, Heart, Myocardial contractile reserve

## Abstract

**Background:**

The C57BL/6J.Y^A/J^ mouse strain is a chromosome-substituted line where the original male-specific portion of chromosome Y (MSY) from C57BL/6J mice was substituted for that from A/J mice. In hearts from male C57BL/6J.Y^A/J^ and C57BL/6J mice, orchidectomy (ORX) affected in a strictly strain-specific fashion the expression a subset of genes showing enrichment for functional categories, including that of circadian rhythms and cardiac contractility. We further tested whether: (1) there were strain-specific differences in cardiac circadian rhythms; (2) strain-dependent differences in the effects of ORX on contractility genes translated into differences in cardiac functions; and (3) differential contractility responses occurred preferentially at times when circadian rhythms also showed strain-specific differences.

**Methods:**

In hearts from the two above strains, we (1) profiled the expression levels of 15 circadian genes at 4-h intervals across a 24 h period; (2) tested the effects of either ORX or androgen replacement on expression of cardiac contractility genes, and that of ORX on myocardial functional reserve; and (3) verified whether the effects of MSY variants on cardiac contractility-related responses showed synchronicity with differences in circadian rhythms.

**Results:**

Among the 15 tested circadian genes, a subset of them were affected by strain (and thus the genetic origin of MSY), which interacted with the amplitude of their peak of maximal expression at 2:00 PM. At that same time-point, ORX decreased (and androgen supplementation increased) the expression of three contractility-related genes, and decreased myocardial relaxation reserve in C57BL/6J.Y^A/J^, but not in C57BL/6J mice. These effects were not detected at 10:00 AM, i.e., at another time-point when circadian genes showed no strain-specific differences.

**Conclusions:**

The results indicate that in mice, androgens have activational effects on cardiac circadian rhythms, contractile gene expression, and myocardial functional reserve. All effects occurred preferentially at the same time of the day, but varied as a function of the genetic origin of MSY. Androgens may therefore be necessary but not sufficient to impart male-specific characteristics to some particular cardiac functions, with genetic material from MSY being one other necessary factor to fully define their range of actions.

**Electronic supplementary material:**

The online version of this article (doi:10.1186/s13293-016-0116-4) contains supplementary material, which is available to authorized users.

## Background

Male-specific characteristics are defined by the actions of androgens (gonadal sex) as well as that of the male-specific portion of chromosome Y (MSY) (chromosomal sex). Although androgens are responsible for many aspects of male differentiation and keep contributing after puberty to many male-specific phenotypes [1, 2], there is emerging evidence that MSY has actions that can be distinguished from that of gonadal steroids [3]. Another manner whereby MSY may affect male-specific characteristics is (as shown previously in rats [4] or mice [5, 6]) via modulation of the effects of androgens, and thus by a mechanism that involves combined actions of both gonadal and chromosomal sex. The clinical importance of these mechanisms relates to the fact that most common diseases display sex-specific differences, with the male sex showing increased cardiovascular risk and vulnerability to related disorders [7, 8]. However, the roles played by androgens in sex-specific cardiovascular differences are not entirely clear, since previous studies showed discrepancies and even sometimes contradictory results [9, 10].

MSY, in addition to possibly having basal effects that manifest themselves in all males, also harbors genetic variants which contribute to phenotypic diversity in traits as varied as cardiovascular functions, immune cell properties, or cancer susceptibility [11, 12]. If genetic MSY variants were to differ in their ability to modulate the effects of androgens, this chromosome might constitute a source of male-specific genetic factors that explain in part why the effects of androgens are not uniform across individuals. However, our understanding of the specific contributions of variants MSY genetic variants has been limited in part by the fact that MSY has structural and functional characteristics that make this chromosomal segment difficult to analyze by genetic techniques that are otherwise standard for other chromosomes [11]. One useful model in this regard has been that constituted by so-called “chromosome-substituted” or “consomic” strains. Among available mouse models, we have previously used the C57BL/6J.Y^A/J/NaJ^ strain (corresponding to C57BL/6J mice in which the original MSY has been substituted for that from A/J mice) for comparisons with the host C57BL/6J strain. These two strains originate from a panel comprising a total of 22 mouse strains, each of which carrying a single chromosome substituted from the donor A/J strain onto a common host C57BL/6J background [13]. Our comparative studies revealed that (1) cardiomyocytes from adult C57BL/6 J.Y^A/J/NaJ^ hearts were smaller than that from their C57BL/6 J counterparts [14]; (2) orchidectomy (ORX) decreased the size of cardiomyocytes from adult C57BL/6J mice, but not that from their C57BL/6 J.Y^A/J/NaJ^ counterparts; and (3) ORX decreased in adult male C57BL/6 J.Y^A/J/Naj^ mice (but not in their C57BL/6J counterparts) the expression of *phospholamban* (*Pln*), titin (*Ttn*) and “four and a half LIM domains 2” (*Fhl2*), i.e., three genes closely related to cardiac contractility, relaxation, and performance [6].

In addition to cardiac contractility, another category that was enriched within genes showing MSY-dependency in their responses to ORX was that of the circadian rhythm signaling pathway [5]. Rhythmic oscillations of circadian genes occur in virtually all organs and tissues, and play important roles in health and disease [15]. Interestingly, circadian rhythms also exhibit sex-related differences [16–18]. In both male and female rodents, gonadal steroids modify the amplitude, free-running period and phase of daily rhythms [17, 19, 20]. In particular, androgens play major roles in the regulation of the suprachiasmatic nucleus, which synchronizes the phase of clocks throughout the body [16, 21]. In hearts, circadian cycles have important functional consequences: (1) in naturally cycling animals, they affect both myocardial metabolism and contractile functions in a coordinate fashion [22], and (2) genetic manipulations that inhibit circadian regulation (either at the whole body level or in a cardiomyocyte-specific fashion) have deleterious effects on cardiac functions, metabolism and signaling properties, and associate with a marked reduction in lifespan [23–25].

To extend our previous studies (which were performed at only one particular time of the day), we compared the expression of circadian genes in hearts of male mice from both strains (either intact or ORX) at 4-h intervals across a 24-h period. Likewise, to verify whether differences in the effects of androgens on cardiac contractility genes translated into functional differences, we compared the effects of either androgen withdrawal or supplementation in hearts from C57BL/6J and C57BL/6J.Y^A/J/NaJ^ male mice. In particular, we tested for potential differences in the effect of androgens on myocardial functional reserve, i.e., a function that is mostly affected by differences in sarcoplasmic reticulum Ca^2+^, which is itself tightly regulated by *Pln* [26]. Since contractility, circadian rhythms and MSY variants may all show interdependent relationships, we tested whether the effects of MSY variants on cardiac functional responses occurred in synchronicity with differential effects on circadian rhythms. We found that the effects of MSY variants on cardiac expression of contractility genes were accompanied by differences in myocardial functional reserve, with all effects occurring preferentially at time-points when circadian genes also showed strain-specific differences. These results, in addition to showing the multiple ways whereby MSY genetic variants may affect cardiac functions, also imply that the activational effects of androgens on some cardiac functions depend in part on the particular genetic makeup of MSY in male mice.

## Methods

### Animals

Male founders for the C57BL/6J and the C57BL/6J.chrY^A/J^ strains (the latter being simply designated C57.Y^A/J^ further in the manuscript) were obtained from The Jackson Laboratory. The strains have been maintained for more than 8 years at the mouse facility of the Institut de recherches cliniques de Montréal (IRCM) by crossing either C57BL/6J or C57BL/6J.chrY^A/J^ males with C57BL/6J females. To minimize the possible occurrence and/or maintenance of *de novo* autosomal variants within the colony, mothers were replaced regularly with females from the original reference genetic stock (obtained from The Jackson Laboratory). Consistent genetic differences between the two strains were thus restricted to just MSY polymorphisms, whose persistence was verified periodically by PCR amplification and sequencing of the genomic region surrounding the MSY rs47889323 single nucleotide polymorphism in animals from each strain, as described previously [6].

All mice were maintained in an animal facility with lights on at 6:00 AM and off at 6:00 PM. All comparisons were made in 12-week-old animals. When ORX mice were needed, surgery was performed at 3 weeks of age in order to prevent the rise of testosterone that normally occurs at puberty, as described previously [5]. Hormonal replacement in ORX animals was performed by subcutaneous implantation of 10-mm-long SILASTIC brand tubing (id, 1.47 mm; od, 1.96 mm; Dow Corning) filled with crystalline 5α-dihydrotestosterone (DHT; Steraloids, Newport, RI) and sealed with silicone 732 Dow Corning rubber glue, as described previously [27]. Implantation was performed in 9-week-old ORX animals, and the animals were killed 3 weeks after the day of implantation. Control animals were implanted with empty SILASTIC tubes of equivalent length. For studies on circadian rhythms, times of the day were defined as “Zeitgeber times”, (ZT), with ZT0 corresponding to 6:00 AM, ZT4 corresponding to 10:00 AM, etc. Starting from ZT0, groups of animals (*n* = 4) were killed at 4-h intervals. During experimental times at night, the animal room was illuminated with red light only.

### Gene expression

Total RNA was extracted from the left ventricular samples and gene expression was monitored by using reverse transcription quantitative polymerase chain reaction (RT-qPCR) to calculate the values of relative expression vs. that of the normalizing *Rps16* housekeeping gene, as described previously [6]. We monitored the expression levels of a total of 15 circadian genes belonging to the following groups: (1) the main circadian clock transcriptional activators (*Bmal1/Arntl*, *Clock* and its *Npas* paralog); (2) the main circadian clock transcriptional repressors (*Per1*, *Per2*, *Per3*, *Cry1*, and *Cry2)*; (3) output genes acting as transcriptional activators and belonging to either the proline and acidic amino-acid-rich basic leucine zipper (PAR bZIP) family (*Dbp*, *Tef*, and *Hlf*) or the retinoic acid related orphan receptor (ROR) family (*Rora*); and (4) output genes acting as transcriptional repressors, i.e., two *Rev-Erb* genes (*Nr1d1* and *Nr1d2*) and the *E4bp4/Nfil3* gene, which belongs to the bZIP family. The composition of each pair of primers is as listed (Additional file 1: Table S1).

### Hemodynamic functional studies

Myocardial functional reserve for either contraction or relaxation was measured in 12-week-old animals as described previously [28]. The mice were anesthetized by intraperitoneal injection of ketamine (100 mg/kg) and xylazine (5 mg/kg), intubated and ventilated, and placed in supine position on a heating pad. A 1.2 F Millar pressure catheter was inserted in the right carotid and pushed into the left ventricles. A central venous line was put in place by inserting into the left jugular vein a PE-10 tubing filled with heparinized saline and connected to a Hamilton syringe. Left ventricular pressure recordings were acquired using a PowerLab/8 SP acquisition system (ADInstruments; Colorado Springs, CO) and combined with electrocardiography signals. After baseline measurements, the saline solution was replaced with one containing dobutamine 50 ng/μl. Administration of dobutamine (1 ng/g/min) was performed by infusing the dopamine solution at a rate of 20 nl/g/min for 4 min, followed by 2 min infusion steps delivering 40, 100, and 200 nl/g/min (i.e., 2, 5 and 10 ng/g/min). Data were analyzed with the Chart for Windows analysis software (ADInstruments). The rates of contraction and relaxation were assessed by recording maximal positive (dP/dt max) and minimal negative (dP/dt min) pressure developments at the end of each incremental infusion step, with the slope of their dose-related changes being used to assess myocardial functional reverse.

### Statistics

The significance of interactions between the effects of strain with that of either time or surgery were calculated by two-way ANOVA, using Prism 7 for Windows (GraphPad Software). Intergroup differences were calculated using post-hoc Tukey tests. Some differences involving only two groups were performed using *t* tests.

## Results

### Strain-dependent differences in cardiac expression of circadian genes

**Fig. 1:**
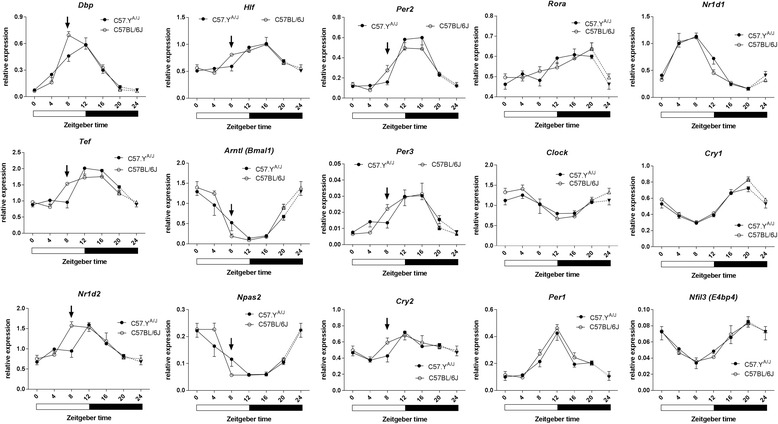
Profiling of expression of 15 circadian genes in hearts from intact C57BL/6J and C57.Y^A/J^ male mice. Expression levels were measured at 4-h intervals between ZT0 and ZT20. Values at each time-point correspond to mean ± SEM (*n* = 4). The *arrows* point to ZT8, which is a time when several circadian genes show some disparity in expression levels. Post-hoc Tukey multiple comparison tests were performed for the three genes where the significance of the strain × time interaction had a *P* value <0.05 (*P < 0.05; **P < 0.01)

**Fig. 2:**
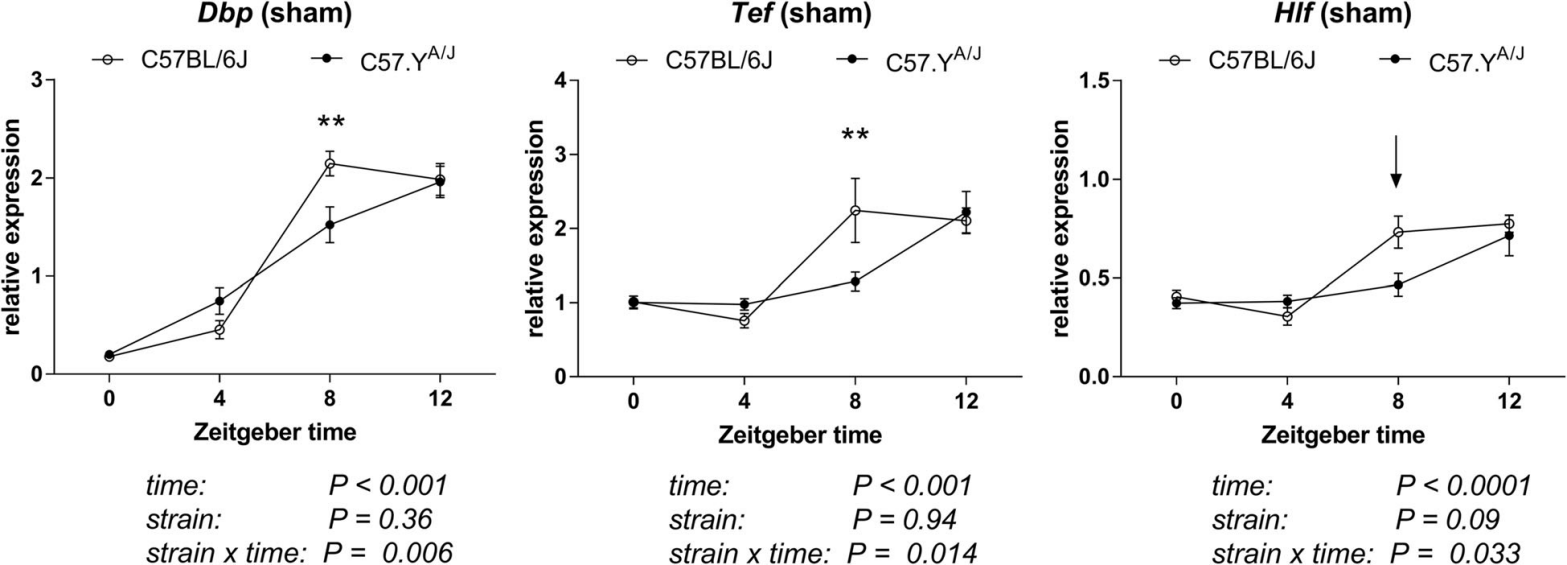
Profiling of expression of *Dbp*, *Tef*, and *Hlf* in hearts from sham-operated C57BL/6J and C57.Y^A/J^ male mice. Expression levels were measured at 4-h intervals between ZT0 and ZT12. Values at each time-point correspond to mean ± SEM (*n* = 7–8). *P* values for each term of the two-way ANOVA analysis are as indicated. The *asterisks* correspond to the significance of the differences detected by post-hoc Tukey multiple comparison tests (**P < 0.01). The *P* values for all terms of the two-way ANOVA tests are as indicated

Across the 24-h period, some circadian genes showed suggestive differences in genes expression, all of them occurring mostly at ZT8 (Fig. 1). For the genes showing the greatest strain-dependent differences, the results of two-way ANOVA analyses of the interactions of strain with time were as follows: *Dbp* (*P* = 0.0148, *F* = 3.1, *η* ^2^ = 0.043), *Tef* (*P* = 0.007, *F* = 3.57, *η* ^2^ = 0.089), and *Nr1d2* (*P* = 0.015, *F* = 3.10, *η* ^2^ = 0.098), with dfn = 6 and dfd = 36 for all groups. None of these interaction terms reached significance when using a Bonferroni-corrected *P* value of 0.003 (i.e., 0.05/15 to correct for the 15 two-way ANOVAs). Nonetheless, we considered the fact that all differences occurred at the same ZT8 time-point to be suggestive, and thus performed a replication experiment with new experimental animals (one full year after the initial experiment). The design of the experiment was also changed in the following manner: (1) a larger number of animals (*n* = 7–8) were used at each time-point; (2) since the previous analysis revealed strain-dependent differences occurred only around ZT8, we restricted our analysis to the daytime points (ZT 0, 4, 8, and 12); and (3) we analyzed only *Dbp*, *Tef* and *Hlf* genes, since the greatest differences were previously observed for the first two genes, and all three correspond to functionally redundant genes that belonged to the same PAR bZIP family [29]. Time (but not strain) had a significant overall effect on expression of all three genes. However, the values of the strain × time interactions were as follows: *Dbp* (*P* = 0.0058, *F* = 4.68, *η* ^2^ = 0.04), *Tef* (*P* = 0.014, *F* = 3.34, *η* ^2^ = 0.09), and *Hlf* (*P* = 0.033, *F* = 3.16, *η* ^2^ = 0.077), with dfn = 3 and dfd = 50 for all groups (Fig. 2). Using a Bonferroni-corrected *P* value of 0.016 (i.e., 0.05/3 to correct for the three two-way ANOVAs), these interactions reached significance for *Dbp* and *Tef*. Of note, these results were very similar to those obtained in the first experiment, with (1) differences being observed at ZT8, and (2) the values of the time × strain interactions being higher for *Dbp* and *Tef* than for *Hlf*. Finally, to test whether the strain-dependent differences were also androgen-dependent, the same experiment with ORX animals was repeated. In the absence of endogenous androgens, interactions of strain with time were no longer observed (Additional file 2: Figure S1).

### Androgen-dependency of expression of contractility-related genes in hearts of C57.Y^A/J^ male mice at ZT8

**Fig. 3:**
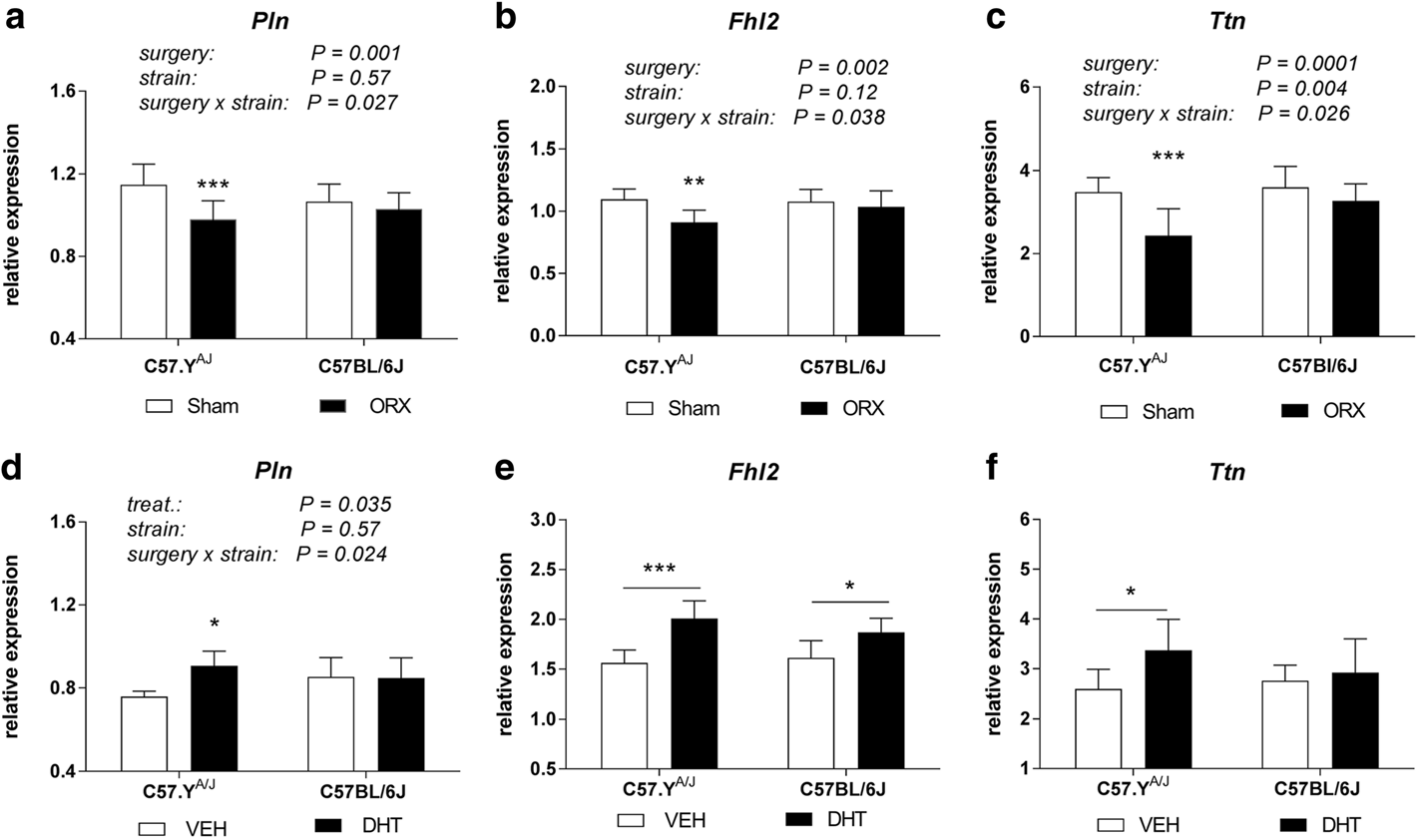
Effects of either surgery (**a**–**c**) or of chronic treatment with DHT (**d**–**f**) on expression levels of *Pln*, *Ttn*, and *Fhl2* in hearts from C57BL/6J and C57.Y^A/J^ male mice. Values at each time-point correspond to mean ± SD (*n* = 6–8). *P* values correspond to those for the interaction of strain with the effect of surgery. The *asterisks* (**a**–**d**) correspond to the significance of the differences detected by post-hoc Tukey multiple comparison tests (*P < 0.05; **P < 0.01; ***P < 0.001). **e**, **f** Significance of differences as detected by *t* tests between animals implanted with either empty (VEH) or DHT-filled (DHT) silastic tubes (*P < 0.05; ***P < 0.001)

We tested the effects of ORX on expression of *Pln, Ttn* and *Fhl2* in hearts of animals killed at ZT8, i.e., at the same time when strain-dependent differences were detected for circadian genes. ORX reduced expression of all three genes in C57.Y^A/J^ mice, but not in that of their C57BL/6J counterparts. Accordingly, there was a significant interaction of the effect of surgery with that of strain for *Pln* (*P* = 0.027, *F* = 5.32, *η* ^2^ = 0.098), *Ttn* (*P* = 0.026, *F* = 5.35, *η* ^2^ = 0.086), and *Fhl2* (*P* = 0.038, *F* = 4.64, *η* ^2^ = 0.237), with dfn = 1 and dfd = 35 for all groups (Fig. 3). In reverse, we also detected a significant interaction of strain with the effect of DHT treatment, with the latter increasing *Pln* expression in hearts from C57.Y^A/J^ mice, but not in that of their C57BL/6J counterparts (*P* = 0.024, *F* = 5.98, *η* ^2^ = 0.19, dfn = 1, dfd = 20). Although the interaction between strain and treatment was not significant by two-way ANOVA for *Fhl2* and *Ttn*, *t* tests revealed that DHT increased expression of these two genes in ORX in C57.Y^A/J^ mice, and that the effect was either less significant (for *Fhl2*) or not detected (for *Ttn*) in C57BL/6J mice (Fig. 3). In animals killed at ZT4, no strain-dependent difference in gene expression was detected across the experimental groups (Additional file 3: Figure S2).

### Strain-specific differences in myocardial functional reserve at ZT8

**Fig. 4:**
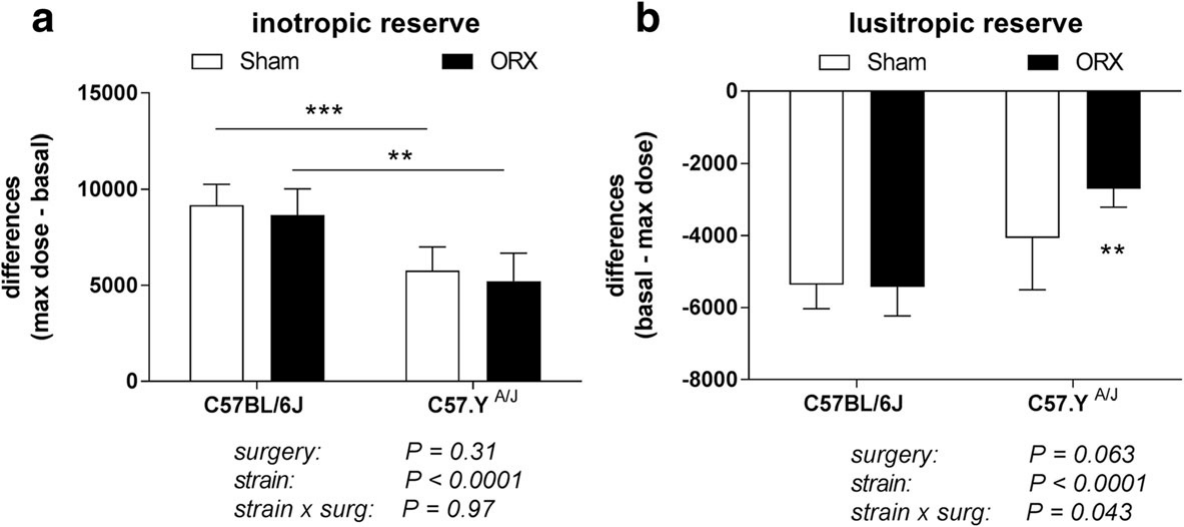
Effects of surgery on myocardial inotropic (*left*) and lusitropic (*right*) reserves. All measurements were performed at ZT8; values are mean ± SD (*n* = 6–8). *Left*: only strain had a significant effect; the *asterisks* correspond to the significance of differences as detected by post-hoc Tukey multiple comparison tests (***P < 0.001; **P < 0.01). *Right*: there was a significant interaction of strain with the effect of surgery on lusitropic reserve. The *asterisks* correspond to the significance of the differences detected by post-hoc Tukey multiple comparison tests (**P < 0.01)

Reduced abundance of *Pln* mRNA typically associates with a reduced sarcoplasmic reticulum Ca^2+^ load [26]. This in turn causes an impairment of myocardial functional reserve, an effect that can be monitored by testing the effects of adrenergic stimulation on cardiac contractility [30, 31]. Dobutamine increased the rates of both contraction and relaxation in a dose-relation fashion, with the magnitude of responses corresponding to myocardial inotropic and lusitropic reserve, respectively. When using doses ranging from 0 to 10 ng/kg/min, we observed that the values of dP/dt max and dP/dt min increased and decreased, respectively, in a linear fashion (Additional file 4: Figure S3). Accordingly, we expressed inotropic reserve as the difference in dP/dt max at the maximal dose of dobutamine *vs*. that recorded at baseline, and lusitropic reserve as the difference in dP/dt min at baseline *vs*. that recorded with the maximal dose of dobutamine. Inotropic reserve was significantly higher in C57BL/6J male mice than their C57.Y^A/J^ counterparts (both in sham-operated and ORX animals), but strain did not interact with the effect of ORX (Fig. 4). In contrast, ORX greatly decreased lusitropic reserve in C57.Y^A/J^ male mice, but not in their C57BL/6J counterparts, causing a significant interaction of strain with the effect of surgery (*P* = 0.043, *F* = 4.65, *η* ^2^ = 0.069). Myocardial functional reserve was not affected by either strain or surgery in animals tested at ZT4 (Additional file 5: Figure S4).

## Discussion

Circadian rhythms, which occur in virtually all organs and tissues, display sex-related differences [16–18] and are affected by gonadal steroids in both male and female rodents. Sex steroids may shape circadian rhythms by acting at several levels and via different mechanisms. Their effects may involve either “organizational” effects (occurring in early development and during puberty) or non-permanent “activational” effects during adulthood [32–34]. One particular study reported that, although the activational effects of gonadal hormones could account for the majority of sex-specific differences in circadian activity, sex chromosome complement could nonetheless play a minor role by affecting the duration of circadian activity bouts [34]. In the current study, we found that ORX affected the peak amplitude of maximal expression of *Dbp* and *Tef* at 2:00 PM in male C57BL/6J mice, but not in their C57.Y^A/J^ counterparts. One possible interpretation of the results is that the increase of expression of *Dbp* and *Tef* that naturally occurs at ZT8 is amplified by physiological levels of androgens in C57BL/6J male mice, but not in their C57.Y^A/J^ counterparts. *Dbp* and *Tef* both belong (along with *Hlf*, whose expression was affected in a similar although non-significant manner) to the PAR bZIP family. They have been reported to be functionally redundant and appear to have cardioprotective properties, as *s*imultaneous deletion of all three leads to the development of cardiac hypertrophy and left ventricular dysfunction [29].

To extend our previous data showing that contractility-related genes were affected by ORX in hearts from C57.Y^A/J^ male mice but not in that of their C57BL/6J counterparts, we further tested whether (1) DHT supplementation affected the expression of these genes in a similar strain-specific fashion and (2) differences in gene expression translated into differences in cardiac functions. These experiments were first performed at ZT8, i.e., at the time when MSY variants were found to affect expression of circadian genes. ORX and DHT supplementation decreased and increased, respectively, the expression the expression of *Pln*, *Ttn*, and *Fhl2* in hearts of male C57.Y^A/J^ mice, but not in that of their C57BL/6J counterparts. The ORX-dependent decrease in the expression of these genes appeared to be functionally important, since ORX also decreased myocardial lusitropic reserve in the same strain-specific fashion. Myocardial functional reserve is mostly affected by differences in sarcoplasmic reticulum Ca^2+^ [30, 31], which is itself tightly regulated by *Pln* and other Ca^2+^ regulatory proteins [26]. Likewise, *Ttn* is a strong modulator of muscle force [35], while *Fhl2* is a regulator of cardiac contractility that also protects the heart against adrenergic stress [36].

The negative effects of ORX on the performance of C57.Y^A/J^ hearts are generally compatible with the intuitive consensus that androgens increase cardiac performance. Although generally true, this concept is supported by only a limited number of animal studies, all of which have been performed in rats. In that species, ORX causes a significant suppression of both contractile and relaxation activities of either isolated hearts [37, 38], myocardial trabeculae [39] or cardiomyocytes [40, 41], with the effects being reversed by testosterone replacement. Interestingly, some reported that while androgens did not always affect basal contractility, they could affect (similarly as found in the current study) myocardial functional reserve [42, 43]. In addition to constituting a useful index that can detect subtle differences in cardiac performance in either humans [30] or animal models [44], cardiac functional reserve also provides dynamic information by measuring the responses of the heart to more challenging conditions [43]. Our data showing that ORX decreases both *Pln* and myocardial functional reserve are also compatible with reports that (1) ORX decreases the amplitude of Ca^2+^ transients and prolongs the time course of Ca^2+^ transient decay, with the effects being reversed by testosterone replacement [41] and (2) androgens increase the expression and/or activity of several Ca^2+^ regulatory proteins that play a predominant role in myocardial contraction and relaxation cycles [26, 39, 42, 45].

Although ORX affected both the expression of contractility genes and myocardial functional reserve in C57.Y^A/J^ hearts at ZT8, no such effects were observed at ZT4, i.e., at a time when cardiac circadian genes showed no strain-dependent differences. We did not demonstrate that the strain-specific effects of ORX on myocardial reserve were a direct consequence of corresponding differences in cardiac circadian gene expression. Of note, it was in C56BL/6J mice where ORX had strain-specific effects on the amplitude of circadian gene expression and (as shown previously [5]) on the size of cardiomyocytes, whereas it was in C57.Y^A/J^ that ORX had strain-specific effects on the expression of contractility genes and myocardial functional reserve. Nonetheless, what is common in all strains is that all effects were detected at ZT8, which may represent a preferential time when MSY variants may affect cardiac functions. This particular time is of particular interest from a cardiovascular stand-point. The sleep-to-wake transition represents a critical time when the heart needs to prepare for changes in energy demands. In humans, the onset of cardiac pathologic events peaks shortly before that time [22]. Mice are nocturnal animals, with their sleep-to-wake transition occurring at ZT12. Interestingly, mouse hearts subjected to ischemia/reperfusion showed greater infarct volume, adverse remodeling and depression of contractile function when the injury was inflicted at ZT12 rather than at ZT0 [46]. We found that MSY variants affected circadian genes expression primarily at ZT8, i.e., the last time-point preceding the critical sleep-to-wake transition. At the very least, the results indicate that (1) the effects of androgens on cardiac performance do not only depend on the genetic make-up of MSY, but that they may also vary in importance at different times across the circadian cycle, and (2) the latter two factors may constitute sources of variance for some of the physiologic effects of androgens.

One implication of our results is that male-specific characteristics of mouse hearts are not shaped by androgens only, but that the effects of the latter also depend on interactions with genetic factors carried by MSY. In rats, it has previously been reported that the effects of MSY variants on blood pressure required the presence of androgen receptors [4] and corresponded to variant-specific interactions of the “sex-determining region on chrY” (*Sry*) gene with androgen receptors [47]. However, rats represent a particular case in terms of MSY genetics because, unlike other vertebrates, it carries 11 copies of *Sry* [47], some of which harbor functional mutations that alter either their temporal expression across tissues or their cellular functions. For instance, the rat genome contains the *Sry2* isoform whose expression is maintained in all tissues and persists during adult life. Likewise, the interaction of *Sry* with the transcriptional activity of androgen receptors depends on a proline to threonine substitution in the rat-specific *Sry3* [47]. Humans carry only one *SRY* copy, with its expression being maintained in a limited set of tissues during adult life [11]. Although human *SRY* interacts with some transcriptional activities of androgens, such interactions have been shown only in the context of prostatic cells [48].

In mice, there is a very tight spatio-temporal control that limits the expression of *Sry* mostly to the precursors of Sertoli cells and at very restricted times during development [49]. We ourselves failed to detect any expression of *Sry* in several mouse adult tissues, including hearts (data not shown). In mice (where it is thus unlikely that the interactions of MSY variants with the effects of androgens involve *Sry*), the exact nature of causal genomic variants thus remains to be identified. Only four MSY protein-coding genes (*Uty*, *Eif2s3y*, *Kdm5d*, and *Ddx3y*) are expressed outside of testes in mice [50]. Although we showed previously that expression of *Uty and Kdm5d* was higher in hearts from C57BL/6J mice than in that from their C57.Y^A/J^ counterparts, these differences were observed only in newly born pups [6]. Nonetheless, such perinatal differences may affect the “organizational” effects of androgens, which themselves may have effects that persist until adulthood. Beyond gene expression, it also remains to be determined to which extent differences in the expression of these genes are matched by differences in the abundance of cognate proteins. For instance, despite being expressed ubiquitously, the *Ddx3y* mRNA transcript is not translated outside of germ cells [51]. One other possibility to consider is that MSY variants may associate with differences in expression of non-protein coding genes (including long non-coding or micro RNAs), although no such genes have been reported yet as originating from mouse MSY. Finally, a full sequence of mouse MSY is currently available for only one strain [52]. More work is therefore needed to determine the exact nature of polymorphisms responsible for the differential effects of MSY variants in mice.

Although it is well accepted that male sex associates with increased cardiovascular mortality, the effects of androgens themselves are more controversial. On one hand, low endogenous bioavailable testosterone levels associate with higher cardiovascular-related mortality, suggesting that androgens may be cardioprotective [9]. On the other hand, while testosterone therapy may have some beneficial cardiac effects, a meta-analysis of placebo-controlled randomized trials also showed that it increased cardiovascular-related events among men [53]. Some have suggested the increased cardiovascular risk in males might relate to factors other than androgens themselves, such as androgen receptor coregulators or determinants of androgen sensitivity [10]. Interestingly, we showed previously that MSY variants associate with differences in the abundance of androgen receptors in the vicinity of genes showing differential responses to ORX [6]. This provides a potential mechanism whereby MSY can govern the sensitivity of particular genes to androgens via changes in chromatin architecture. Moreover, MSY might be considered as a source of male-specific genetic factors explaining in part why the effects of androgens vary across individuals. A better understanding of how MSY variants modulate the effects of androgens and which genes are responsible for such effects may improve our assessment of male-specific risk by helping to predict whether androgens will have either positive or negative effects in particular individuals, or to identify which ones are most likely to be either affected negatively by declines in testosterone and/or benefit from androgen supplementation.

## Conclusions

Although androgens are necessary to impart male-specific characteristics to cardiac functions, they may not be sufficient to shape some of such functions, with the concomitant presence of genetic factors carried by MSY being required to fully define the range of their effects. Moreover, genetic variants of MSY vary in their ability to modulate the activational effects of androgens. Their effects are sufficiently important to have an impact on cardiac functions, and may possibly explain some lack of consistency in previously published concerning the effects of androgens on the heart.

## Additional files

Additional file 1: Table S1. Primers used for RT-qPCR analysis. (PDF 168 kb)
Additional file 2: Figure S1. Profiling of expression of Dbp, Tef, and Hlf in hearts from ORX C57BL/6 J and C57.YA/J male mice. Expression levels were measured at 4-h intervals between ZT0 and ZT12. Values at each time-point correspond to mean ± SEM (*n* = 7–8). Unlike sham-operated animals, there was no interaction between strain and time. (PDF 62 kb)
Additional file 3: Figure S2. Effects of surgery on expression of Fhl2 and Pln in heart from either C57BL/6 J or C57YA/J male mice. In contrast to ZT8, no significant difference was detected at ZT4. (PDF 45 kb)
Additional file 4: Figure S3. Linear regressions of the values of either dP/dt max (left0 or dP/dt min (right) at doses of dobutamine ranging from 0 to 10 ng/kg/min. All measurements were obtained in sham-operated C57BL/6 J or C57.YA/J male mice (*n* = 6–8). The asterisks correspond to the significance values of multiple comparison Tukey post-hoc tests (**P* < 0.05; ***P* < 0.01; *****P* < 0.0001). (PDF 64 kb)
Additional file 5: Figure S4. Effects of surgery on myocardial inotropic (left) and lusitropic (right) reserves in animals tested at ZT4. Values are mean ± SD (*n* = 6–8). In contrast to animals tested at ZT8 (see Fig. 4), no significant differences were detected between the groups. (PDF 48 kb)

## Abbreviations

DHT: Dihydrotestosterone
MSY: Male-specific portion of chromosome Y
ORX: Orchidectomy or orchidectomized
RT-qPCR: Reverse transcription quantitative polymerase chain reaction
ZT: Zeitgeber time
