# Supplementary material for: Comparisons of chromosome Y-substituted mouse strains reveal that the male-specific chromosome modulates the effects of androgens on cardiac functions
Source: Biol Sex Differ. 2016 Nov 23;7:61. doi: 10.1186/s13293-016-0116-4 (PMC5143463; doi:10.1186/s13293-016-0116-4)
Supplement: Additional file 1: Table S1. — Primers used for RT-qPCR analysis. (PDF 168 kb) [file 13293_2016_116_MOESM1_ESM.pdf]

Table S1: Primers used for RT-qPCR analysis

Gene symbol	Forward primer	Reverse primer
Arntl/Bmal1	CTATTACCAGGGCAGGCTCA	TTGCTGCCTCATCGTTACTG
Clock	TCCATCCAGTATGCCACAGA	CCCACAAGCTACAGGAGCAG
Cry1	GTGGGAAGCGTCCTAGTCAG	ACCAACTTCAGTTGCGAGGA
Cry2	CCATCGTCAATCATGCAGAG	CCACACAGGAAGGGACAGAT
Dbp	AGGAACTGAAGCCTCAACCA	TGGCTGCTTCATTGTTCTTG
Fhl2	CCATTGGAACCAAGAGCTTC	TGCTCCCGGTAAGTAACACC
Hlf	GCTCGCAAAGTCTTCATTCC	CGATCTGGTTCTCCTTCAGC
Nfil3/(E4bp4)	GTTACAGCCGCCCTTTCTTT	CATCCATCAATGGGTCCTTC
Npas2	GCCCTCAGAAGTCAGCAGAA	TGCTGTTGGTAGGGTGTGAG
Nr1d1	CTTCCGTGACCTTTCTCAGC	TCACTGTCTGGTCCTTCACG
Nr1d2	CTGGCTTCCGAGATCTGTCT	ACAGTCCGTTCCTTTGCATC
Per1	CTGGGGACCAGGTCATTAAG	CTTCAGCACAGAGGCTGCAT
Per2	TTCGGCACATCCCAAAGT	CTCCGTGTCTGGGATCATTT
Per3	AGAAGCTCCAGAGCATGGAA	TCTGTCTTCACAGGCGACAC
Pln	CACTGTGACGATCACCGAAG	TTTCCATTATGCCAGGAAGG
Rora	GAACCACCGAGAAGATGGAA	GGAAAATGGAGTCGCACAAT
Rps16	GCTACCAGGGCCTTTGAGATG	AGGAGCGATTTGCTGGTGTGG
Tef	GTTTGCAGAGGAGGACCTGA	GAGCGTTTAGCTGCCACATT
Ttn	CACACAGCCCAGATCTCAAA	TTCTGGAGCGACTCACACTG
