# Supplementary material for: Comparisons of chromosome Y-substituted mouse strains reveal that the male-specific chromosome modulates the effects of androgens on cardiac functions
Source: Biol Sex Differ. 2016 Nov 23;7:61. doi: 10.1186/s13293-016-0116-4 (PMC5143463; doi:10.1186/s13293-016-0116-4)
Supplement: Additional file 2: Figure S1. — Profiling of expression of Dbp, Tef, and Hlf in hearts from ORX C57BL/6 J and C57.YA/J male mice. Expression levels were measured at 4-h intervals between ZT0 and ZT12. Values at each time-point correspond to mean ± SEM (n = 7–8). Unlike sham-operated animals, there was no interaction between strain and time. (PDF 62 kb) [file 13293_2016_116_MOESM2_ESM.pdf]

Fig. S1

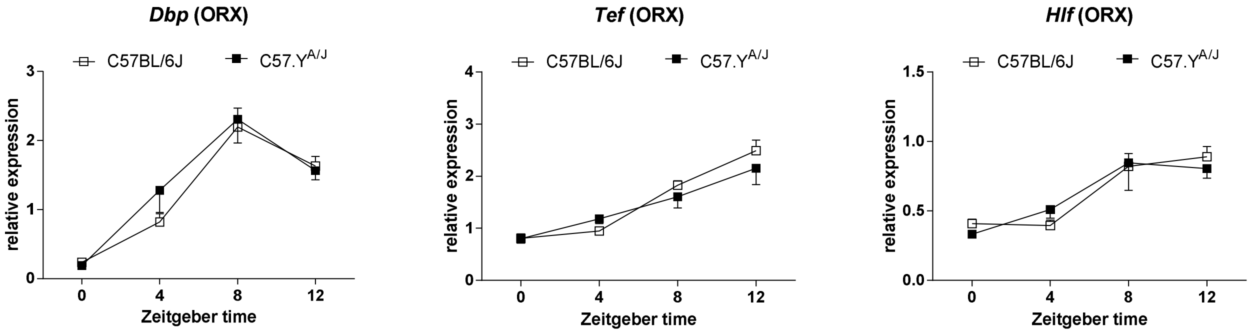

Fig. S1: Profiling of expression of *Dbp*, *Tef* and *Hlf* in hearts from ORX C57BL/6J and C57.$Y^{A/J}$ male mice. Expression levels were measured at 4-hour intervals between ZT0 and ZT12. Values at each time-point correspond to mean ± SEM (n = 7-8). Unlike sham-operated animals, the was no interaction between strain and time.
