# Supplementary material for: Comparisons of chromosome Y-substituted mouse strains reveal that the male-specific chromosome modulates the effects of androgens on cardiac functions
Source: Biol Sex Differ. 2016 Nov 23;7:61. doi: 10.1186/s13293-016-0116-4 (PMC5143463; doi:10.1186/s13293-016-0116-4)
Supplement: Additional file 3: Figure S2. — Effects of surgery on expression of Fhl2 and Pln in heart from either C57BL/6 J or C57YA/J male mice. In contrast to ZT8, no significant difference was detected at ZT4. (PDF 45 kb) [file 13293_2016_116_MOESM3_ESM.pdf]

Fig. S2

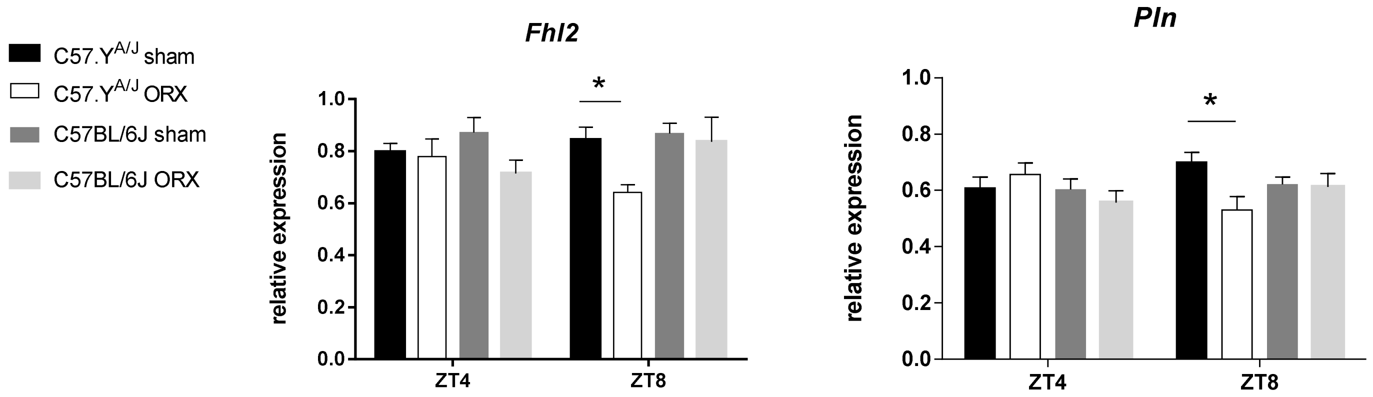

Fig. S2: Effects of surgery on expression of Fhl2 and Pln in heart from either C57BL/6J or C57YA/J male mice. In contrast to ZT8, no significant difference was detected at ZT4.
