# Supplementary material for: Comparisons of chromosome Y-substituted mouse strains reveal that the male-specific chromosome modulates the effects of androgens on cardiac functions
Source: Biol Sex Differ. 2016 Nov 23;7:61. doi: 10.1186/s13293-016-0116-4 (PMC5143463; doi:10.1186/s13293-016-0116-4)
Supplement: Additional file 4: Figure S3. — Linear regressions of the values of either dP/dt max (left0 or dP/dt min (right) at doses of dobutamine ranging from 0 to 10 ng/kg/min. All measurements were obtained in sham-operated C57BL/6 J or C57.YA/J male mice (n = 6–8). The asterisks correspond to the significance values of multiple comparison Tukey post-hoc tests (*P < 0.05; **P < 0.01; ****P < 0.0001). (PDF 64 kb) [file 13293_2016_116_MOESM4_ESM.pdf]

Fig. S3:

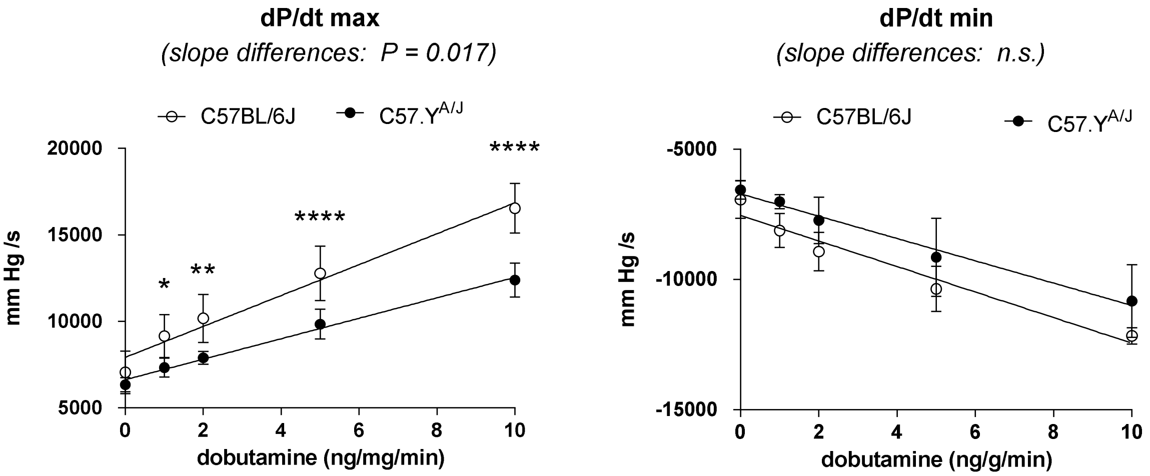

Fig. S3: **Linear regressions of the values of either dP/dt max (left0 or dP/dt min (right) at doses of dobutamine ranging from 0 to 10 ng/kg/min**. All measurements were obtained in sham-operated C57BL/6J or C57.$Y_{A/J}$ male mice (n = 6-8). The asterisks correspond to the significance values of multiple comparison Tukey's post-hoc tests (*$P < 0.05$; **$P < 0.01$; ****$P < 0.0001$).
