# Supplementary material for: Comparisons of chromosome Y-substituted mouse strains reveal that the male-specific chromosome modulates the effects of androgens on cardiac functions
Source: Biol Sex Differ. 2016 Nov 23;7:61. doi: 10.1186/s13293-016-0116-4 (PMC5143463; doi:10.1186/s13293-016-0116-4)
Supplement: Additional file 5: Figure S4. — Effects of surgery on myocardial inotropic (left) and lusitropic (right) reserves in animals tested at ZT4. Values are mean ± SD (n = 6–8). In contrast to animals tested at ZT8 (see Fig. 4), no significant differences were detected between the groups. (PDF 48 kb) [file 13293_2016_116_MOESM5_ESM.pdf]

Fig. S4:

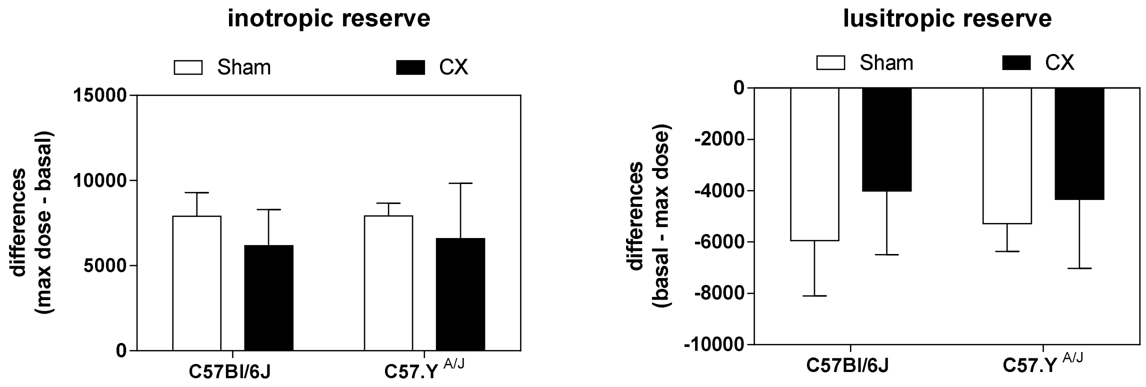

Fig. S4: Effects of surgery on myocardial inotropic (left) and lusitropic (right) reserves in animals tested at ZT4. Values are mean ± SD (n = 6-8). In contrast to animals tested at ZT8 (see Fig. 5), no significant differences were detected between the groups.
